# High Neutrophil-Lymphocyte Ratio, Platelet-Lymphocyte Ratio and Low Lymphocyte Levels Are Correlated With Worse Pathological Complete Response Rates

**DOI:** 10.7759/cureus.22972

**Published:** 2022-03-08

**Authors:** Serdar Karakaya, İbrahim Karadağ, Mehmet Emin Yılmaz, Ömür Berna Çakmak Öksüzoğlu

**Affiliations:** 1 Medical Oncology, Health Science University, Atatürk Chest Diseases and Chest Surgery Training and Research Hospital, Ankara, TUR; 2 Department of Medical Oncology, Çorum Hittite University Erol Olçok Training and Research Hospital, Çorum, TUR; 3 Department of Internal Medicine, Health Sciences University, Ankara Dr. Abdurrahman Yurtaslan Oncology Training and Research Hospital, Ankara, TUR; 4 Department of Medical Oncology, Health Sciences University, Ankara Dr. Abdurrahman Yurtaslan Oncology Training and Research Hospital, Ankara, TUR

**Keywords:** neoadjuvant chemoradiotherapy, rectum cancer, platelet-lymphocyte ratio, pathological response, neutrophil-lymphocyte ratio

## Abstract

Objective: To investigate the effect of hemogram parameters on predicting pathological complete response (pCR) in locally advanced rectal cancer.

Methodology: A total of 227 patients with rectal cancer treated with neoadjuvant concurrent chemoradiotherapy (CRT) were retrospectively analyzed. All patients were divided into two subgroups as high or low hemogram parameters according to the cut-off value obtained using the receiver operating characteristic (ROC) curve.

Results: In patients with low neutrophil-lymphocyte ratio (NLR) and platelet-lymphocyte ratio (PLR) levels, pCR rate was statistically significantly higher than the group with high NLR and PLR levels (for NLR: 39.77% vs. 5.34%; p<0.001, for PLR: 32.38% vs 7.01%; p<0.001 respectively). In addition, the pCR rate was significantly better in patients with high lymphocyte levels compared to the group with low lymphocyte levels (33.33% vs. 7.5%; p<0.001, respectively). According to the multivariate logistic regression analysis result, NLR and PLR levels were considered as independent predictors to predict pathological complete response [p<0.001, HR: 0.128 (95% CI=0.051 - 0.322) for NLR; p=0.017, HR: 0.332 (95% CI=0.134 - 0.821) for PLR, respectively].

Conclusion: Our study showed that high NLR, PLR, and low lymphocyte levels were correlated with worse pCR rates. In addition to that, NLR and PLR emerged as independent predictive markers.

## Introduction

Colorectal cancer is the third most common malignancy and ranks second among cancer deaths [1]. Rectal cancer accounts for nearly one-third of all colorectal cancers, and almost half of them are diagnosed with locally advanced stage [2]. In Turkey, according to official statistics, 41% of colorectal cancers were found to be locally advanced [3]. The standard treatment approach in locally advanced rectal cancer is neoadjuvant concurrent chemoradiotherapy (CRT) followed by total mesorectal excision (TME) after six to eight weeks of waiting, and treatment outcomes have improved remarkably in recent years [4,5]. There are both long-term and short-term CRT applications. On the other hand, the total neoadjuvant approach, which has come to the fore with the RAPIDO and PRODIGE 23 studies recently, has short- and long-term results such as higher pathological complete response (pCR) and longer disease-free survival (DFS) than the standard treatment arm, especially in T4 and node-positive patients [6,7]. With these results, total neoadjuvant approach stands out as a new treatment approach. Many studies, including the NSABP R-04 study, show that patients who received concomitant CRT before surgery can achieve a pCR rate of 16-22% [8]. Yet, there is limited information about which patients can get a complete response.

Inflammation-based blood biomarkers play an important role in cancer evolution, especially in tumorigenesis and tumor progression [9]. It has been found in previous studies that the behavior of the tumor could be predicted by systemic inflammatory biomarkers [10]. It has been suggested that biomarkers such as neutrophil to lymphocyte ratio (NLR) and platelet to lymphocyte ratio (PLR), which are inexpensive, easily accessible, and useful systemic inflammatory biomarkers, could be used as prognostic factors in many types of cancer by revealing the interaction between host immune status and inflammation [11]. It has also been documented in numerous cancer types that high NLR and PLR levels are poor prognostic factors [12-14]. Moreover, it has been reported that high NLR and PLR levels in rectal cancer and other cancers would adversely impact both the pathological response and the radiological response [5,15-18].

In our study, we planned to investigate the function of systemic inflammatory biomarkers NLR and PLR in predicting pathological complete response in patients receiving neoadjuvant concurrent chemoradiotherapy. Thus, we aimed to predict which patients would have a high probability of achieving a pathological complete response before treatment.

## Materials and methods

Patients

In this study, 1300 colorectal cancer patients followed in a single oncology center between 2015 and 2021 were retrospectively screened. Four hundred fifty of these patients were diagnosed with rectal cancer, and 227 patients who met the inclusion criteria were included in the study. Patients over the age of 18, diagnosed with locally advanced rectal cancer, receiving standard neoadjuvant concurrent chemoradiotherapy, and patients who could be operated on after neoadjuvant therapy were included in the study. Patients with secondary malignancies, patients under 18 years of age, those with additional comorbidities (such as diabetes, chronic obstructive pulmonary disease, heart failure), those who did not receive the treatment completely, and those with conditions that may impact systemic inflammatory markers such as active infection, chronic inflammatory or autoimmune disease, and steroid use were excluded from the study. Along with the demographic data of all patients, their height, body surface areas, and complete blood count parameters were recorded at the time of diagnosis. NLR and PLR were calculated with the formula: Neutrophil count (/µL) / Lymphocyte count (/µL) and Platelet count (10^9^/L) / Lymphocyte count (/µL).

Treatment regimen

All patients received radiotherapy in 25-28 fractions at a total dose of 45-50 Gy. Concurrent capecitabine chemotherapy was administered at a dose of 825 mg/m2 twice a day on the days of radiotherapy. After concurrent CRT, the patients were operated on after six to eight weeks of convalescence. All patients received adjuvant chemotherapy.

Pathological evaluation

Postoperative histopathological evaluation was noted, and staging was performed using the American Joint Committee on Cancer (AJCC) Staging Manual (7th edition) [19]. Tumor regression grades (TRG) were recorded according to the AJCC 7th edition as follows: TRG0 - no residual tumor cells; TRG1 - single cells or small groups of cells; TRG2 - residual cancer but predominant fibrosis behind; and TRG3 - minimal or no tumor response. Those with TRG0 are defined as the 'pCR group'; whereas TRG1, TRG2 and TRG3 were defined as the 'non-pCR group' [20].

Statistical analysis

Statistical analysis was performed via the software of SPSS 25.0 (IBM Corp., Armonk, NY, USA). Mann-Whitney U test was used for comparison of nonparametric data, and Student T-test was used for comparison of parametric data. Chi-Square or Fisher's Exact test was used for comparison of categorical data. Prognostic factors impacting pCR were identified by conducting multivariate analysis with the Cox proportional hazards model. The results were considered statistically significant at p<0.05. Receiver operating characteristic (ROC) analysis was used for the pCR of lymphocyte, NLR, and PLR in predicting treatment response, and the value closest to the point with the maximum sensitivity and specificity was selected as the optimal cut-off value. In the study, we determined the primary endpoint as pCR. Approval from the local ethics committee of Health Sciences University, Ankara Dr. Abdurrahman Yurtaslan Oncology Training and Research Hospital, was obtained for our study with the decision number 2022-01/1565 on 26.01.2022.

## Results

The median age of diagnosis was 60 (IQR 51-68 years) and 58.1% (n=132) of the patients were male. In the surgery performed after neoadjuvant concurrent CRT, 19.2% (n=42) of the patients had a complete response. Some patient characteristics are defined in Table 1.

**Table 1:** Baseline patients characteristics APR, abdominal perineal resection; LAR: low anterior resection

Variables		
Age (median) min-max		60 (26-88) years
Gender	Male (n)	132 (58.1%)
	Female (n)	95 (41.9%)
Preoperative Stage	2	96 (42.3%)
	3	74 (32.6%)
	Not evaluable	57 (25.1%)
Preoperative lymph node number with MRI	none	24 (10.6%)
	1	9 (4%)
	>1	163 (71.8%)
	Missing data	31 (13.7%)
Operation type	APR	56 (24.7%)
	LAR	98 (43.2%)
	Missing data	73 (32.2%)

Mean NLR and PLR levels were determined to be significantly lower in the pCR group compared to the non-pCR group (p1<0.001, p2<0.001, respectively). On the other hand, mean lymphocyte level was found to be significantly higher in the group with pCR compared to the group with non-pCR (p<0.001). There was no significant difference between the two groups in terms of mean neutrophil and platelet counts. The results related to hematological sub-parameters are summarized in Table 2. The group with pCR and the groups with non-pCR were similar in terms of stages (p=0.814).

**Table 2:** Comparison of hemogram parameters in pCR group and non-pCR group NLR: neutrophil-lymphocyte ratio; PLR: platelet-lymphocyte ratio, pCR: pathological complete response

	pCR group	Non-pCR group	P value
NLR (mean±SD)	2.27±0.56	3.7934±2.71	<0.001
PLR (mean±SD)	153.35±50.19	4.65±2.03	<0.001
Lymphocyte count(10^3^)	1.995±0.67	1.490±2.03	<0.001
Neutrophil count (10^3^)	4.35±1.43	4.65±2.03	0.372
Platelet count (10^3^)	285.75±92.55	290.09±96.88	0.797

**Figure 1:**
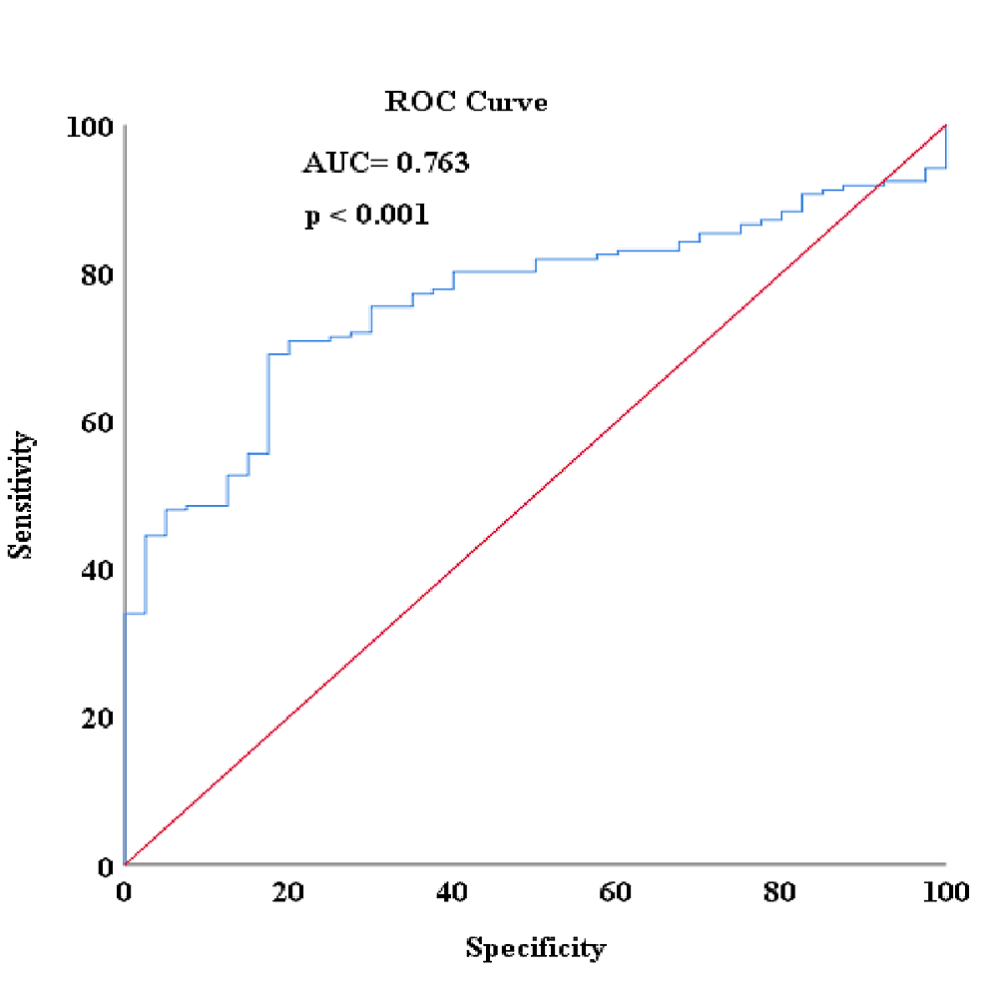
Detecting non-pCR with NLR score in ROC analysis NLR: neutrophil-lymphocyte ratio; pCR: pathological complete response; ROC: receiver operating characteristic

**Figure 2:**
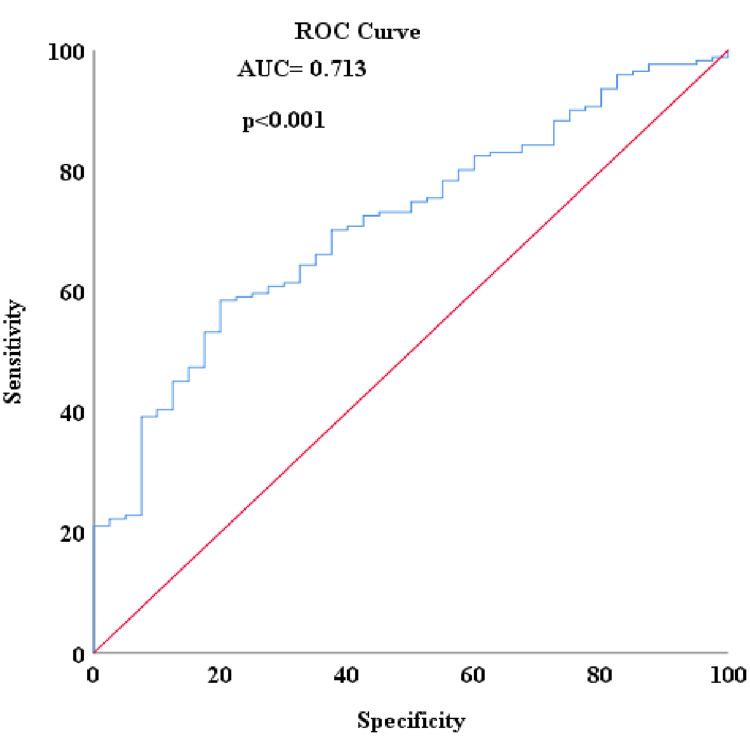
Detecting non-pCR with PLR score in ROC analysis PLR: platelet-lymphocyte ratio; pCR: pathological complete response; ROC: receiver operating characteristic

**Figure 3:**
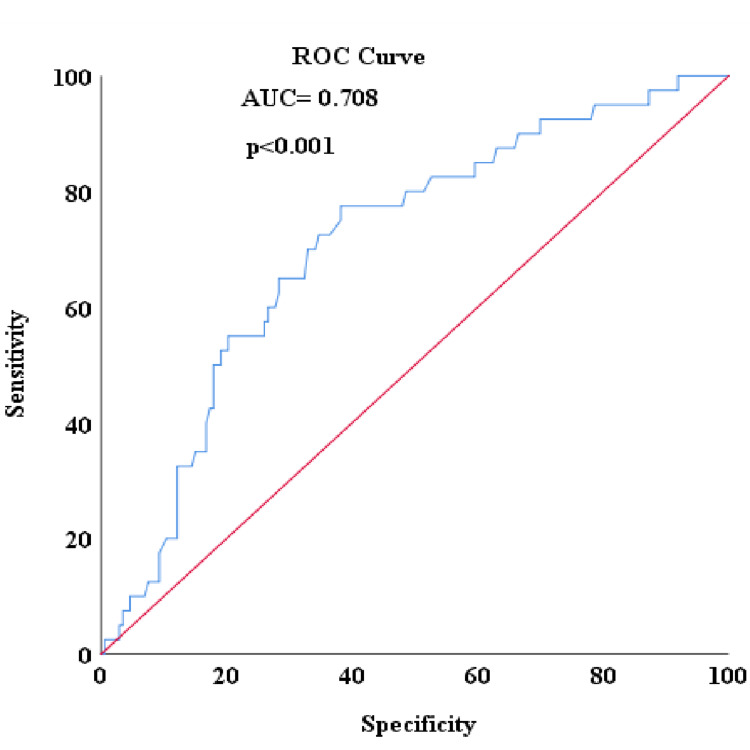
Detecting non-pCR with lymphocyte count in ROC analysis pCR: pathological complete response; ROC: receiver operating characteristic

When ROC analysis was conducted to detect patients with no complete response in the study, the area under the curve (AUC) for NLR was 0.763, with an ideal cut-off value of 2.56 corresponding to an optimum sensitivity of 70.8% and a specificity of 80% (Figure 1). The pCR rate was higher in patients with low NLR levels compared to the group with high NLR levels (39.77% vs. 5.34%; p<0.001, respectively). For the PLR, the AUC was determined to be 0.713, with an ideal cut-off value of 180.50, corresponding to an optimum sensitivity of 58.8% and a specificity of 80% (Figure 2). The pCR rate was found to be significantly higher among patients with low PLR levels compared to the group with high PLR levels (32.38% vs 7.01%; p<0.001, respectively). For the lymphocyte level, the AUC was 0.708, with an ideal cut-off value of 1580, corresponding to optimum sensitivity of 77.5% and specificity of 61.8% (Figure 3). The pCR rate was significantly higher in patients with high lymphocyte levels compared to the group with low lymphocyte levels (33.33% vs. 7.5%; p<0.001, respectively).

When multivariate logistic regression analysis was conducted with NLR, PLR and lymphocyte level, NLR and PLR levels were considered as independent predictive markers in predicting pathological complete response [p<0.001, HR: 0.128 (95% CI=0.051 - 0.322) for NLR; p=0.017, HR: 0.332 (95% CI=0.134 - 0.821) for PLR, respectively] (Table 3).

**Table 3:** Multivariate analysis of the effect of NLR, PLR and lymphocyte levels NLR: neutrophil-lymphocyte ratio; PLR: platelet-lymphocyte ratio

	HR (95%CI)	P value
NLR		
Low	Reference	<0.001
High	0.128 (0.051 – 0.322)	
PLR		
Low	Reference	0.017
High	0.332 (0.134 – 0.821)	
Lymphocyte		
Low	Reference	0.365
High	1.580 (0.588-4.247)	

## Discussion

In this study, we demonstrated that the pCR rate was higher in low NLR and PLR values and high lymphocyte levels. Besides, we also revealed that NLR and PLR were independent predictive markers for predicting pCR.

Various factors have been investigated as potential predictors of response to neoadjuvant concurrent CRT in locally advanced rectal cancer. However, the number of studies on the prediction of treatment response is limited [21-24]. Inflammatory response is closely associated with the development of tumors, and increased inflammatory response is associated with poor prognosis in many cancers [25]. It has been shown in numerous studies that various systemic inflammatory markers in rectal cancer receiving neoadjuvant CRT are independent prognostic factors [26,27]. The results of our study suggest that the relationship systemic inflammation indices could be used in predicting the CRT response and pathological complete response in patients with rectal cancer receiving concurrent CRT. There are several other studies suggesting that systemic inflammatory indices such as NLR and PLR are predictors of the pathological response to neoadjuvant CRT [15-16,28-29]. In the presented study, unlike other studies, it has been shown that lymphocyte level can also be a marker in predicting pCR [24]. From our point of view, the high number of patients in this study may have made a difference in obtaining this result [24]. Moreover, in our study, similar to other studies, NLR and PLR were independent predictive factors after adjustment for confounding factors [15,16].

NLR consists of a combination of neutrophils and lymphocytes, and anti-tumor activity and activation of the inflammatory response impact this ratio [30,31]. Low NLR levels are associated with higher tumor response [30,32]. Like NLR, PLR is also a considerable marker of the inflammatory response, and anti-tumor efficacy and treatment response is better at low PLR levels in locally advanced rectal cancer [17,29]. It is well-documented that low lymphocyte levels reduce anti-tumor activity [33]. In the present study, it was found that NLR and PLR could be used to predict pathological complete response and it was also demonstrated that lymphocyte levels could contribute to this prediction.

When administering neoadjuvant concurrent CRT, one of our primary goals is to achieve a pathological complete response. It is well-known that the success of local control increases by achieving a pathological complete response even if there is no difference in overall survival by achieving a pathological complete response [34]. Some studies have demonstrated a survival benefit with pCR [35]. In the light of these studies, it is well known that failure to achieve pathological complete response worsens oncological outcomes. Since it may be less likely to achieve pCR, especially in the patient group with high pre-treatment NLR and PLR levels, the total neoadjuvant approach, which is now emerging as the new standard treatment regimen, can be a considerable option in this patient group [6,7]. Hence, we believe that it may be more beneficial to prioritize total neoadjuvant treatment regimens in patients with high pre-treatment blood NLR and PLR. These findings can be supported by further research.

The presented study's major limitations are that this is a single-centered and retrospective study; thus, it has missing data. On the other hand, the study reflects real-life data with a substantial number of patients, even though it is a single-center study, which stands out as an advantage.

## Conclusions

In conclusion, in our study we were able to demonstrate that high blood NLR, PLR, and low lymphocyte levels were correlated with worse pCR rates. In addition to that, NLR and PLR emerged as independent predictive markers. The total neoadjuvant treatment approach should be strongly considered in patients with high NLR and PLR levels. Further prospective studies with a larger population are needed to prove these findings.

## References

[1] Ke TM, Lin LC, Huang CC, Chien YW, Ting WC, Yang CC (2020). High neutrophil-to-lymphocyte ratio and platelet-to-lymphocyte ratio predict poor survival in rectal cancer patients receiving neoadjuvant concurrent chemoradiotherapy. Medicine (Baltimore).

[2] Wasserberg N (2014). Interval to surgery after neoadjuvant treatment for colorectal cancer. World J Gastroenterol.

[3] (2019). Republic of Turkey Ministry of Health, General Directorate of Public Health 2016 Turkey Cancer Statistics Report 2019: Page 33. https://sbu.saglik.gov.tr/Ekutuphane/kitaplar/health-statistics-yearbook-2019pdf.pdf.

[4] Guillem JG, Chessin DB, Cohen AM (2005). Long-term oncologic outcome following preoperative combined modality therapy and total mesorectal excision of locally advanced rectal cancer. Ann Surg.

[5] Andras D, Crisan D, Craciun R (2020). Neutrophil-to-lymphocyte ratio: a hidden gem in predicting neoadjuvant treatment response in locally advanced rectal cancer?. J BUON.

[6] Bahadoer RR, Dijkstra EA, van Etten B (2021). Short-course radiotherapy followed by chemotherapy before total mesorectal excision (TME) versus preoperative chemoradiotherapy, TME, and optional adjuvant chemotherapy in locally advanced rectal cancer (RAPIDO): a randomised, open-label, phase 3 trial. Lancet Oncol.

[7] Conroy T, Bosset JF, Etienne PL (2021). Neoadjuvant chemotherapy with FOLFIRINOX and preoperative chemoradiotherapy for patients with locally advanced rectal cancer (UNICANCER-PRODIGE 23): a multicentre, randomised, open-label, phase 3 trial. Lancet Oncol.

[8] Allegra CJ, Yothers G, O'Connell MJ (2014). Neoadjuvant therapy for rectal cancer: mature results from NSABP protocol R-04. J Natl Cancer Inst.

[9] Pease NA, Wise-Draper T, Privette Vinnedge L (2015). Dissecting the potential interplay of DEK functions in inflammation and cancer. J Oncol.

[10] Morrison L, Laukkanen JA, Ronkainen K, Kurl S, Kauhanen J, Toriola AT (2016). Inflammatory biomarker score and cancer: a population-based prospective cohort study. BMC Cancer.

[11] Klevorn LE, Teague RM (2016). Adapting cancer immunotherapy models for the real world. Trends Immunol.

[12] Shibutani M, Maeda K, Nagahara H (2013). A high preoperative neutrophil-to-lymphocyte ratio is associated with poor survival in patients with colorectal cancer. Anticancer Res.

[13] Jung MR, Park YK, Jeong O, Seon JW, Ryu SY, Kim DY, Kim YJ (2011). Elevated preoperative neutrophil to lymphocyte ratio predicts poor survival following resection in late stage gastric cancer. J Surg Oncol.

[14] Erdur E, Yildirim OA, Poyraz K, Aslan F, Yıldız F, Kömek H (2021). The role of inflammatory parameters in predicting disease recurrence in patients with stage IIA colon cancer with no high-risk features. Postgrad Med.

[15] Sun Y, Huang Z, Chi P (2020). An inflammation index-based prediction of treatment response to neoadjuvant chemoradiotherapy for rectal mucinous adenocarcinoma. Int J Clin Oncol.

[16] Li A, He K, Guo D, Liu C, Wang D, Mu X, Yu J (2019). Pretreatment blood biomarkers predict pathologic responses to neo-CRT in patients with locally advanced rectal cancer. Future Oncol.

[17] Kim TG, Park W, Kim H (2019). Baseline neutrophil-lymphocyte ratio and platelet-lymphocyte ratio in rectal cancer patients following neoadjuvant chemoradiotherapy. Tumori.

[18] Karakaya S, Karadağ İ, Ateş Ö, Çakmak Öksüzoğlu ÖB (2021). Can neutrophil-to-lymphocyte ratio or platelet-to lymphocyte ratio predict chemotherapy response in testicular cancer?. Eurasian J Med Investig.

[19] Mace AG, Pai RK, Stocchi L, Kalady MF (2015). American Joint Committee on Cancer and College of American Pathologists regression grade: a new prognostic factor in rectal cancer. Dis Colon Rectum.

[20] Kim SH, Chang HJ, Kim DY (2016). What is the ideal tumor regression grading system in rectal cancer patients after preoperative chemoradiotherapy?. Cancer Res Treat.

[21] Garcia-Aguilar J, Chen Z, Smith DD (2011). Identification of a biomarker profile associated with resistance to neoadjuvant chemoradiation therapy in rectal cancer. Ann Surg.

[22] Chan J, Kinsella MT, Willis JE (2013). A predictive genetic signature for response to fuoropyrimidine-based neoadjuvant chemoradiation in clinical Stage II and III rectal cancer. Front Oncol.

[23] Dayde D, Tanaka I, Jain R, Tai MC, Taguchi A (2017). Predictive and prognostic molecular biomarkers for response to neoadjuvant chemoradiation in rectal cancer. Int J Mol Sci.

[24] Eraslan E, Adas YG, Yildiz F, Gulesen AI, Karacin C, Arslan UY (2021). Systemic immune-inflammation index (SII) Predicts pathological complete response to neoadjuvant chemoradiotherapy in locally advanced rectal cancer. J Coll Physicians Surg Pak.

[25] Diakos CI, Charles KA, McMillan DC, Clarke SJ (2014). Cancer-related inflammation and treatment effectiveness. Lancet Oncol.

[26] Yamamoto A, Toiyama Y, Okugawa Y (2019). Clinical implications of pretreatment: lymphocyte-to-monocyte ratio in patients with rectal cancer receiving preoperative chemoradiotherapy. Dis Colon Rectum.

[27] Yang J, Xu H, Guo X, Zhang J, Ye X, Yang Y, Ma X (2018). Pretreatment inflammatory indexes as prognostic predictors for survival in colorectal cancer patients receiving neoadjuvant chemoradiotherapy. Sci Rep.

[28] Krauthamer M, Rouvinov K, Ariad S (2013). A study of inflammation-based predictors of tumor response to neoadjuvant chemoradiotherapy for locally advanced rectal cancer. Oncology.

[29] Ramsay G, Ritchie DT, MacKay C, Parnaby C, Murray G, Samuel L (2019). Can haematology blood tests at time of diagnosis predict response to neoadjuvant treatment in locally advanced rectal cancer?. Dig Surg.

[30] Dudani S, Marginean H, Tang PA (2019). Neutrophil-to-lymphocyte and platelet-to-lymphocyte ratios as predictive and prognostic markers in patients with locally advanced rectal cancer treated with neoadjuvant chemoradiation. BMC Cancer.

[31] Braun LH, Baumann D, Zwirner K (2019). Neutrophil-tolymphocyte ratio in rectal cancer-novel biomarker of tumor immunogenicity during radiotherapy or confounding variable?. Int J Mol Sci.

[32] Jeon BH, Shin US, Moon SM, Choi JI, Kim MS, Kim KH, Sung SJ (2019). Neutrophil to lymphocyte ratio: a predictive marker for treatment outcomes in patients with rectal cancer who underwent neoadjuvant chemoradiation followed by surgery. Ann Coloproctol.

[33] Gooden MJ, de Bock GH, Leffers N, Daemen T, Nijman HW (2011). The prognostic influence of tumour-infiltrating lymphocytes in cancer: a systematic review with meta-analysis. Br J Cancer.

[34] Ryan R, Gibbons D, Hyland JM (2005). Pathological response following long-course neoadjuvant chemoradiotherapy for locally advanced rectal cancer. Histopathology.

[35] Shia J, Guillem JG, Moore HG (2004). Patterns of morphologic alteration in residual rectal carcinoma following preoperative chemoradiation and their association with long-term outcome. Am J Surg Pathol.

